# Components of well-being and distress that foster resilience in medical students

**DOI:** 10.1192/j.eurpsy.2024.501

**Published:** 2024-08-27

**Authors:** A. Eőry, K. T. Radvanyi, S. Kudrinko, G. Vajda, S. Rózsa, M. Kellermayer

**Affiliations:** ^1^Family Medicine; ^2^Semmelweis Univerity; ^3^Personality and Health Psychology, Károli Gáspár University of the Reformed Church; ^4^Biophysics and Radiation Biology, Semmelweis Univerity, Budapest, Hungary

## Abstract

**Introduction:**

The shortage of medical professionals is becoming a nearly unsurmountable burden worldwide. Increasing uncertainties in the external environment require enhanced capacity for predicting future outcomes from the young adult population of medical students.

**Objectives:**

To find the level of resilience, the domains of psychological well-being and the symptoms of distress; and to identify associations between them in a cohort of medical students.

**Methods:**

Data were collected in the 2022-23 academic year among Hungarian and English medical students at Semmelweis University. An online questionnaire was circulated via the official academic administration system (Neptun) with the incentive to provide personal results with available resources for those who requested it. Besides age and gender, we applied the short form of the Nicholson McBride Resilience Questionnaire (NMRQ), Ryff’s Psychological Well-being Scales (PWB), and the Depression, Anxiety and Stress Scale (DASS). Additional to descriptive statistics, univariate analyses as well as multiple regression analyses (SPSS v.24) were used. (Ethics permission No: BM/5326-2/2023).

**Results:**

Altogether 318 students (132 Hungarians) filled the questionnaire, and 251 students requested personal answers. 114 males participated with a mean age slightly higher than that of females (24 (SD:4) ys vs 23 (SD:3) ys). Hungarians (H) demonstrated lower resilience (Mdn:37, IQR:32, 42) then international (I) students (Mdn: 41, IQR: 36, 47), U=15287, p<0.001. Both H and I students showed similar patterns on PWB, scoring highest on personal growth, purpose in life and positive relations with others; while reaching lowest scores on environmental mastery, and lower scores on autonomy and self-acceptance. However, they demonstrated differences in each of the domains (see table).

**Table:** 

	Hungarian n=132 (Mn (SD))	International n=186 (Mn (SD))	t (316)	p
Autonomy (A)	34 (7.9)	36 (7.4)	-2.8	0.004
Environmental Mastery (EM)	32 (8.4)	34 (7.7)	-2,1	0.04
Personal Growth (PG)	42 (5.6)	40 (7.2)	2.9	0.004
Positive Relations (PR)	41 (8.4)	37 (7.7)	4.6	<0.001
Purpose in Life (PL)	42 (7.8)	38 (8.0)	3.5	<0.001
Self-Acceptance (SA)	36 (10.4)	36 (7.7)	0.2	n.s.

Higher prevalence of symptoms of depression, anxiety and stress were found in I students. Multiple regression analyses resulted in statistically significant models for both H (F(11, 121)=19.6; p<0.001; R^2^=0.641) and I students (F(12, 143)=8.98; p<0.001; R^2^=0.430) indicating that EM (t=4.7; p<0.001), PL (t=--3.2; p=0.002), SA (t=4.2; p<0.001), A (t=2.9; p=0.005), and anxiety (t-4.06; p<0.001) significantly predicted the strength of resilience in H, while autonomy (t=4.9; p<0.001) proved to be significant predictor in case of I students.

**Conclusions:**

These single-centre results need to be further clarified on national and international level to stimulate interventions for strengthening resilience through establishing a caring network by universities for the fragile population of medical students.

**Disclosure of Interest:**

None Declared

